# Evidence-Based Community Health Worker Program Addresses Unmet Social Needs And Generates Positive Return On Investment

**DOI:** 10.1377/hlthaff.2019.00981

**Published:** 2020-02

**Authors:** Shreya Kangovi, Nandita Mitra, David Grande, Judith A. Long, David A. Asch

**Affiliations:** Division of General Internal Medicine, Perelman School of Medicine, University of Pennsylvania, in Philadelphia.; Department of Epidemiology and Biostatistics, Perelman School of Medicine, University of Pennsylvania.; Division of General Internal Medicine, Perelman School of Medicine, University of Pennsylvania.; Division of General Internal Medicine, Perelman School of Medicine, University of Pennsylvania.; Division of General Internal Medicine, Perelman School of Medicine, University of Pennsylvania.

## Abstract

Interventions that address socioeconomic determinants of health are receiving considerable attention from policy makers and health care executives. The interest is fueled in part by expected returns on investment. However, many current estimates of returns on investment are likely overestimated, because they are based on pre-post study designs that are susceptible to regression to the mean. We present a return-on-investment analysis that is based on a randomized controlled trial of Individualized Management for Patient-Centered Targets (IMPaCT), a standardized community health worker intervention that addresses unmet social needs for disadvantaged people. We found that every dollar invested in the intervention would return $2.47 to an average Medicaid payer within the fiscal year.

Each year the United States spends roughly $550 billion on care for the nearly sixty-three million Americans covered by Medicaid^1^—which accounts for one-sixth of national health care spending.^2^ Some of this spending may be inefficient because it is used to treat illnesses as they manifest themselves, instead of addressing the underlying social and behavioral factors that cause illness.^3^ To maximize health and the value of spending, policy makers such as officials in the Department of Health and Human Services are encouraging health care organizations to experiment with interventions that address upstream social determinants of health.^4,5^ Social determinants of health are the conditions in which people live and work—including income, social relationships, and education.^6^ A growing number of health care organizations have hired community health workers (trusted individuals from local communities) to provide social support, care coordination, and advocacy for high-risk patients. Several studies have described sociobehavioral interventions delivered by community health workers that are effective in improving clinical outcomes such as chronic disease control,^7,8^ mental health,^9^ quality of care,^10^ and hospital use.^8,10-12^

The growing interest in community health worker programs is fueled in part by expected cost savings.^13^ However, with few exceptions,^12,14,15^ these programs have not been subjected to rigorous economic analysis. Two systematic reviews^12,14^ noted that most evaluations of community health worker programs for adult patients either lack adequate cost data or suffer from design limitations—especially the use of pre-post study designs that are susceptible to regression to the mean. Helen Jack and co-authors^12^ noted that all seven of the pre-post studies they reviewed that assessed hospitalization (a key driver of costs) showed decreased rates of hospitalization in the post period. However, six of the seven randomized controlled trials reviewed showed no decrease in hospitalization rates. Thus, there is a risk of overestimating cost savings from reductions in hospitalization rates unless economic analyses are based on well-designed clinical trials. The two reviews^12,14^ collectively identified only one randomized controlled trial of a community health worker program that included cost data—a study that focused on a pediatric population.^16^

Here we present an economic analysis of Individualized Management for Patient-Centered Targets (IMPaCT), a standardized community health worker intervention^8,11,17-20^ that addresses socioeconomic and behavioral barriers to health in low-income populations. It has been studied in three prior randomized controlled trials,^8,11,21^ including a recent trial we conducted among chronically ill, uninsured, or Medicaid-insured outpatients^8^ showing that IMPaCT improved glycosylated hemoglobin, body mass index, tobacco cessation, mental health, and quality of care and reduced hospitalizations.

In this article we use outcomes data from this randomized controlled trial^8^ to calculate a return on investment for the program from the perspective of a Medicaid payer.

## Study Data And Methods

### OVERVIEW

The IMPaCT intervention^8,11,17-19,22^ was tested in a randomized controlled trial (hereafter referred to as “the trial”)^8^ that enrolled 302 patients (150 randomly assigned to the intervention arm and 152 to the control arm) between July 12, 2013, and October 15, 2014. In this article we use outcomes data collected in the trial (the number and acuity of inpatient admissions and charges for them, as well as the number of outpatient visits) to estimate cost savings generated by the intervention. We present annualized expenses, cost savings, and return on investment for an average team of six community health workers serving 330 patients per year.

### INTERVENTION DETAILS

IMPaCT is a theory-based^17^ intervention in which specially hired and trained community health workers provide tailored social support for high-risk patients. There are varying durations and intensities of IMPaCT depending on population needs. The trial upon which this economic analysis was based tested a six-month, high-intensity program among 302 adult patients who were insured by Medicaid or uninsured, were residents of high-poverty neighborhoods, and had been diagnosed with at least two chronic diseases (diabetes, obesity, tobacco dependence, or hypertension). The trial did not require that patients have a prior hospitalization or otherwise be predicted to incur high costs to be enrolled.

After enrollment, community health workers used a semistructured interview guide^17,19^ to learn patients’ life stories and understand their social needs (such as housing instability, food insecurity, and limited social support). This conversation informed tailored, patient-driven action plans. Over the next six months community health workers communicated weekly with patients and helped them execute their action plans. For example, one patient told her community health worker that she ate unhealthy food to cope with family stress, and she wanted to find a more healthy, creative outlet. The community health worker helped her enroll in a pottery class at a local senior center. Beyond their one-on-one work with patients, community health workers also led a weekly support group intended to create social networks among high-risk patients. Indeed, one of the important aspects of this intervention is that community health workers targeted social and recreational activities and not just the pathways of conventional medicine, such as help with medication adherence. However, community health workers were closely integrated with outpatient primary care practices (they had work space in the practice and access to the electronic medical record) and coordinated their efforts with those of clinical staff. They sent electronic messages to clinical staff at regular intervals, communicating patients’ action plans and progress. They also used ad hoc electronic messages, telephone calls, or meetings for any clinical matters (for example, a patient who was running out of medications).

### INFRASTRUCTURE

Community health worker programs vary in their structure, and that variation likely affects program costs and effects. IMPaCT is highly structured and includes recommended caseloads; supervision ratios; hiring algorithms; training courses; and software for documentation, reporting, and quality control.

At the time of the trial, IMPaCT community health workers were already full-time employees of the study health system, operating in an existing large-scale program that serves approximately 2,000 patients a year—including trial participants.^18^ (In other words, the workers included in the trial were already health system employees and were not hired just for the trial.) Since the trial was pragmatic, study participants were incorporated into community health workers’ routine work flows and caseloads.

The study health system centralizes its community health worker program instead of relying on each practice or hospital to hire, train, and supervise the workers. Managers—typically people with a master’s degree in social work or public health—supervise teams of six community health workers who each serve 55 patients annually, for a collective caseload of 330 patients. A coordinator supports the workers by helping identify and enroll patients and collecting data for ongoing quality assurance; one coordinator can serve two manager-led teams. A program director oversees the health system’s entire community health worker program (eight manager-led teams) and is responsible for high-level operations, including budgets, hiring, and quality assurance. (See online appendix 1 for a figure that shows team structure and caseloads.)^23^

Community health workers on any given manager-led team can be deployed into various practices or hospitals across the health system, where they work closely with clinical teams as noted above. This centralization allows for economies of scale: Practices that can support only one or two community health workers benefit from a robust infrastructure.

### PROGRAM EXPENSES

Annual program expenses were calculated for a team of six community health workers delivering the IMPaCT outpatient intervention to 330 unique patients. We included expenses for all infrastructural program elements, including 2018 salaries and benefits for the community health workers, manager, director, and coordinator. We included the proportion of director and coordinator effort spent on any one team.We also included the cost of equipment, public transportation for community health workers, and office space (which was minimal because community health workers are mobile). In addition, we included discretionary expenses for patients that community health workers are able to request from managers case by case (for example, vouchers for cab fares or rapport-building activities). The program also pays for YMCA memberships so community health workers can exercise or attend classes with patients.We also added indirect costs (using a 12 percent indirect rate, typical for community-based organizations without clinical or laboratory equipment) to cover overhead costs.^24^

### PROGRAM OUTCOMES

During the randomized controlled trial we obtained Social Security numbers for trial participants and sent them to the Pennsylvania Healthcare Cost Containment Council (which operates a statewide database for hospital discharge records across Pennsylvania).^25^ Based on these identifiers, the council provided us with the total number of allcause general acute care inpatient admissions across the commonwealth for trial participants within one year of trial enrollment. The council’s database also included diagnosis-related group (DRG) and total hospital facility charges for each admission. Importantly, this database aggregates hospital discharge records, not claims. Therefore, it includes only charges (that is, the “list price” that hospitals charge payers), not the amounts actually paid. Charges can vary substantially from the amounts actually paid by insurers such as Medicaid, even when cost-to-charge ratios are applied.^26^

Separately, we also extracted data on the number of outpatient visits from the study health system’s electronic medical records.

In the trial we used negative binomial regression to test differences in the total count of admissions, charges, and outpatient visits by study arm.

### MEDICAID COST AND ACUITY DATA

In our primary analysis we used the number and acuity of inpatient admissions and outpatient visits to estimate the costs by paid by Medicaid for patients who received the intervention compared to control patients.We conducted a secondary analysis to calculate total inpatient charges for intervention versus control patients, to explore whether differences in charges were similar to differences in cost from our primary analysis.

State policy determines whether Medicaid pays for inpatient care by admission (based on DRGs) or by bed day with per diem payments.^27^ In Pennsylvania, where the trial was conducted, Medicaid pays by admission. So for our primary analysis, to estimate inpatient costs, we used the 2018 average Medicaid facility fee payment per admission reported by the Pennsylvania Health Care Cost Containment Council^28^ and increased it by 17.7 percent to reflect typical Medicaid professional fees as a percentage of facility fees.^29^ (Appendix 3 contains an analysis using bed days, for readers in per diem states.)^23^

We adjusted this inpatient admission cost estimate to reflect the acuity of admissions as follows. First, we estimated the acuity of each admission by multiplying its DRG code by standardized case-mix weights published by the Centers for Medicare and Medicaid Services.^30^ We averaged these case-mix weights by study arm and divided them by the average case-mix weight for all Medicaid discharges in our data set. We multiplied these arm-specific adjustment factors by the Medicaid cost per admission to arrive at an acuity-adjusted average cost of admission for each arm.

To estimate the cost of outpatient visits, we used the mean cost of a level 3 office visit (the most common type of office visit).^31^ We used a level 3 office visit instead of using actual acuity-adjusted outpatient visit levels for simplicity, because outpatient visit acuity levels have a minimal impact on cost.

### FINANCIAL ANALYSIS

We first calculated the difference between inpatient costs for intervention versus control patients. To do this, we started with raw data from the trial: the number of admissions per patient-year for intervention patients.We scaled this by 330 (an average team’s caseload), which yielded the total number of intervention admissions per year for a team of community health workers. We multiplied this number by the acuity-adjusted cost of an admission for a Medicaid payer in Pennsylvania to arrive at the total inpatient costs for patients of a team of community health workers. We applied the same logic to calculate inpatient costs for the same number of control patients, and we then calculated the difference in inpatient costs between intervention and control patients to arrive at inpatient costs saved by the intervention.

Similarly, we calculated total outpatient costs for intervention and control patients. The difference between these costs was the excess cost for outpatient visits that was attributable to a team of community health workers per year. We subtracted the excess outpatient cost attributable to the community health workers from the inpatient savings to estimate the total annual savings realized by a team of workers. We calculated the return on investment by dividing the savings realized by one team of community health workers by the expenses incurred by that team.

In our secondary analysis we calculated the reduction in total inpatient charges for intervention versus control patients.

### LIMITATIONS

This study had several limitations. First, the trial was not powered for hospital or outpatient utilization. However, we have seen similar effect sizes from all three randomized controlled trials^8,11,22^ of this intervention. To address uncertainty, we conducted a two-way sensitivity analysis that simultaneously varied the estimated differences in hospital and outpatient utilization attributable to the intervention by 25 percent in either direction.

Second, our database also had certain limitations. The hospitalization data were limited to general acute care admissions and did not include psychiatric or skilled nursing facility stays, emergency department visits, or any pharmacy information. We also did not include other supplemental payments linked to, but not directly associated with, an individual inpatient hospital service—including payments for teaching or safety-net hospitals. This could have led to an underestimation of inpatient cost estimates.

Third, the outpatient utilization data were limited to the study health system. However, Medicaid patients must pick a primary care provider and can switch only after contacting their insurer. This likely limits the degree to which patients use out-of-system outpatient visits.

Fourth, we present expenses of a program that is arguably operating at optimal scale. It took the study health system one year to ramp up to the optimum, where long-run average cost is minimized. This relatively rapid ramp-up was facilitated by the use of standardized tools such as hiring algorithms, training, and software.

Finally, 82 percent of participants in the trial had Medicaid insurance, while the remainder were uninsured. In post hoc subgroup analyses we found that the effectiveness of the intervention was the same for patients with both insurance types. Thus, in this economic analysis from the perspective of a Medicaid payer, we assumed that Medicaid was the payer for all patients.

## Study Results

### PROGRAM EXPENSES AND OUTCOMES

Total annual expenses for a full team were $567,950.82 (exhibit 1).

At baseline, there were no significant differences between the study arms in hospitalizations before enrollment in the study. At one year after enrollment, 31.6 percent of patients in the control arm had been hospitalized, compared to 23.3 percent of those in the intervention arm (*p* = 0.11) (data not shown). The 152 patients in the control arm had a total of 98 admissions during the one-year follow-up period, or 0.64 admissions per patient-year (exhibit 2). The 150 patients in the intervention arm had 68 admissions, or 0.45 per patient-year—a 30 percent relative reduction (*p* = 0.17).

Control patients had higher-acuity admissions than intervention patients did. The average DRG case-mix weight was 1.38 for control patients, 1.21 for intervention patients, and 1.31 for all patients (data not shown). Thus, the acuity adjustment factor was 1.05 for control patients and 0.92 for intervention patients (exhibit 2).

Patients in the control arm had a mean of 11.4 outpatient visits per year, compared with 12.2 in the intervention arm (*p* = 0.57).

### RETURN ON INVESTMENT TO A MEDICAID PAYER

The average facility cost to a Medicaid payer for an admission was $14,000, which we increased to $16,478 to reflect the addition of professional fees (exhibit 2).

The intervention arm had both fewer and lower-cost admissions, with a total inpatient cost of $2,267,900.10, compared with $3,681,206.88 in the control arm. When outpatient costs were factored in, the total cost of care was $2,450,881.80 for the intervention arm and $3,852,189.78 for the control arm; thus, the intervention resulted in a 38 percent reduction in cost.

Overall, a team of community health workers saved Medicaid $1,401,307.99. This savings divided by program expenses ($567,950.82) yielded a return of $2.47 for every dollar invested, realized within a single fiscal year. In a sensitivity analysis that varied the number of admissions and outpatient visits attributable to the intervention, we found that the return ranged from $1.84 to $3.09 (see appendix 2).^23^

### SECONDARY ANALYSIS

The goal of our secondary analysis was to explore whether the reduction in inpatient charges by study arm was similar to what we calculated using average Medicaid cost. The total inpatient charges at one year after enrollment for patients in the intervention arm was $3,897,124, compared with $6,365,699 in the control arm—a reduction of 39 percent (*p* = 0.76) (data not shown). This effect size was similar to the 36 percent reduction in cost from our primary analysis.

## Discussion

Within a single fiscal year the standardized, evidence-based, Individualized Management for Patient-Centered Targets community health worker program yielded an annual return of $2.47 for every dollar invested, from the perspective of a Medicaid payer. To our knowledge, this is the first economic analysis of a health system–based community health worker intervention for adults that used data from a randomized controlled trial. This is significant because the analysis provided a more realistic estimate of the return on investment, compared with estimates derived from pre-post evaluations that are likely to be exaggerated because of regression to the mean (that is, reductions in spending that are not attributable to an intervention but rather to random variation).

Policy makers and health care organizations interested in making similar investments in community health worker programs should interpret this study in the context of four key points.

First, effectiveness and, consequently, the return on investment are determined by the specific intervention’s characteristics and should be extrapolated to other community health worker programs with caution. IMPaCT has several characteristics that may drive its effectiveness. For instance, unlike many “screen-and-refer” approaches to addressing unmet social needs,^32,33^ IMPaCT is a theory-based intervention with an emphasis on personalized action plans and hands-on support. It also has a robust and standardized infrastructure. Implementation science studies of global community health worker programs demonstrate that insufficient investment in infrastructure or unrealistic caseloads can compromise program effectiveness.^34-36^ Thus, light-touch programs with insufficient infrastructure can appear cheaper initially but ultimately waste resources.

Second, the financial value of a program depends on the baseline costs among the targeted patient pool. For this reason, many programs that address social determinants are offered only to patients predicted to incur high costs. At our health system we wanted to ensure that IMPaCT was available for patients who were considered at high risk for poor health not only by virtue of frequent hospitalizations but also by other measures (for example, a patient with a glycosylated hemoglobin of 12 who never goes to the hospital). This article demonstrates that even when applied to this broader population, the community health worker intervention returned $2.47 for every dollar invested.

Third, return on investment depends critically on who is making the investment and who is receiving the return. We have presented an economic analysis from the perspective of a Medicaid payer, assuming that the payer bears all costs and receives all returns. In reality, providers often bear some of the costs for community health worker programs and see returns only if costs of prevented admissions exceed revenue (for example, uncompensated care for uninsured patients), beds opened up by prevented admissions can be “backfilled” with other patients who generate even more favorable margins, or providers receive incentive payments for meeting quality targets and containing costs. In the case of the study health system, issues of who pays and who benefits have been internalized by the negotiation of joint funding for the IMPaCT program from a regional Medicaid managed care organization and the provider health system. However, these agreements are challenging to negotiate, and failure to align who pays and who benefits will likely lead to underinvestment in community health worker programs.

Fourth, this study suggests that IMPaCT is beneficial, even from a narrow business perspective. That said, the financial return on investment underestimates the true social return because any cost-based measure of effectiveness overemphasizes the value of avoiding hospitalization (which is expensive) relative to improving health (which is often financially silent). Interventions that increase recommended cancer screening,^37^ facilitate lead testing in children, or identify patients with hypertension through community outreach^38^ can enormously advance health even as they remain invisible to systems that measure only charges that flow through accounting systems with one-year time horizons. Even accountable care drivers, which seemingly focus on value, typically focus on the value seen on balance sheets. In contrast, patients measure value in units that are almost always off the books.

## Implications

We have described a community health worker model that achieves a favorable return on investment for Medicaid payers by effectively responding to the social determinants of health. Our pragmatic return-on-investment analysis has influenced a regional Medicaid payer to expand its investments from the delivery of patient care, which is directly reimbursed, to the delivery of social support—which was previously not reimbursed but which nevertheless adds health and financial value. We believe that the same calculations are likely to be relevant to other populations, providers, and insurers. ■

## Supplementary Material

Supplementary Material

## Figures and Tables

**EXHIBIT 1:** Program expenses for a team of Individualized Management for Patient-Centered Targets (IMPaCT) community health workers including supervision and infrastructure, in one fiscal year

Expense	Amount
Personnel	
Six community health workers	$307,550.06
Supervision and support (manager, coordinator, director)	146,666.98
Personnel subtotal	454,217.04
Equipment and services	
Smartphones and service	6,739.20
Laptops	13,805.19
Ongoing training	3,530.00
Weekly team meetings	1,434.00
Patient expenses	2,500.00
Transportation for the community health worker	6,732.00
YMCA memberships	2,250.00
Office supplies	1,821.15
Equipment subtotal	38,811.54
Office space rent	14,070.36
Direct costs total	507,098.94
Indirect rate	12%
Yearly total	$567,950.82
Cost per patient for 6-month intervention	$1,721.06

**source** Authors’ analysis of financial data from the study health system.

**EXHIBIT 2:** Calculation of inpatient and outpatient costs for intervention versus control patients and return on investment for a team of Individualized Management for Patient-Centered Targets (IMPaCT) community health workers (CHWs), in one fiscal year

	Number
Estimated total (facility and professional) Medicaid cost per admission	$16,478
Number of patients per CHW team	330
Inpatient costs for intervention patients	
Admissions per patient-year	0.45
Admissions per CHW team	149.6
DRG case-mix weight for admissions	0.92
Case-mix-adjusted per admission cost for admissions	$15,159.76
Total inpatient costs for intervention patients	$2,267,900.10
Inpatient costs for control patients	
Admissions per patient-year	0.64
Admissions per CHW team	212.76
DRG case-mix weight for admissions	1.05
Case-mix-adjusted per admission cost for admissions	$17,301.90
Total inpatient costs for control patients	$3,681,206.88
Inpatient cost savings	$1,413,306.79
Outpatient costs for intervention patients	
Outpatient visits per patient-year	12.2
Outpatient visits per CHW team	4,026
Average Medicaid cost per outpatient visit	$45
Total outpatient costs for intervention patients	$182,981.70
Outpatient costs for control patients	
Outpatient visits per patient-year	11.4
Outpatient visits per CHW team	3,762
Average Medicaid cost per outpatient visit	$45
Total outpatient costs for control patients	$170,982.90
Excess outpatient costs	$11,998.80
Estimated Medicaid savings per year	$1,401,307.99
Return on investment	$2.47

**sources** Pennsylvania Healthcare Cost Containment Council and authors’ analysis. **notes** Numbers might not add to totals because of rounding. The return on investment is the estimated Medicaid savings divided by expenses per team. DRG is diagnosis-related group.
