# Supplementary material for: Evidence-Based Community Health Worker Program Addresses Unmet Social Needs And Generates Positive Return On Investment
Source: Health Aff (Millwood). Author manuscript; Available in PMC 2021 Nov 3. (PMC8564553; doi:10.1377/hlthaff.2019.00981)
Supplement: Supplementary Material [file NIHMS1748829-supplement-Supplementary_Material.pdf]

Kangovi S, Mitra N, Grande D, Long JA, Asch DA. Evidence-based community health worker program addresses unmet social needs and generates positive return on investment. Health Aff (Millwood). 2020;39(2).

Appendix 1: Infrastructure for an IMPaCT team

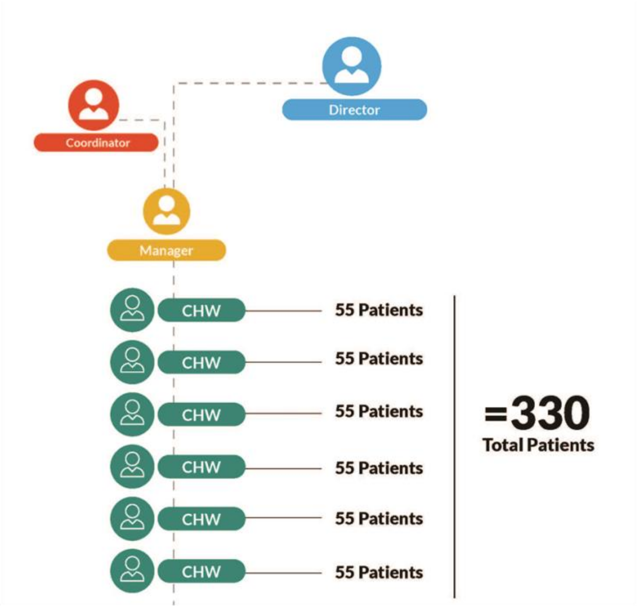

Appendix 1: Infrastructure for a CHW team. CHWs work in teams of six serving 330 patients annually. Teams are supervised by a manger (typically an MSW or MPH). A coordinator helps to identify and enroll patients, and collecting data for ongoing quality assurance. One coordinator can serve two manager-led teams. A high-level director supervises eight manager-led teams.

**Appendix 2: Sensitivity Analysis**			
	**Base Case**	**Best Case (inpatient savings inflated by 25% and additional outpatient costs decreased by 25%)**	**Worst Case (inpatient savings lowered by 25% and additional outpatient costs increased by 25%)**
**Inpatient Savings**	$1,413,307	$1,766,633	$1,059,980
**Additional Outpatient Costs**	$11,999	$8,999	$14,998
**Total Medicaid Savings Per Year**	1,401,308	1,757,634	1,044,982
**ROI based on cost savings and expense calculation**	2.47	3.09	1.84

**Appendix 3: Cost Calculation Using Bed Days**	
**Assumptions**	
Estimated Total Medicaid Cost Per Bed Day (Facilities and Professional Fees)	$2,738
Number of Patients (Caseload for a CHW Team)	330
**Inpatient Costs for Intervention Patients**	
Number of Bed-Days Per Patient-Year for Intervention Patients	1.85
Total Number of Bed-Days Per Team for Intervention Patients	611.6
DRG Case-Mix Weight for Intervention Admissions	0.92
Estimated Total (Facilities and Professional) Medicaid Cost Per Bed-Days	$2,738
Case Mix Adjusted Cost for Control Intervention Bed-Days	$2,518.69
**Total Inpatient Costs for Intervention Patients**	**$1,540,428.26**
**Inpatient Cost for Control Patients**	
Number of Bed-Days Per Patient-Year for Control Patients	2.72
Total Number of Bed-Days Per Team for Control Patients	898.82
DRG Case-Mix Weight for Control Admissions	1.05
Estimated Total (Facilities and Professional) Medicaid Cost Per Bed-Days	$2,738
Case Mix Adjusted Cost for Control Group Bed-Days	$2,874.59
**Total Inpatient Costs for Control Patients**	**$2,583,724.27**
**Inpatient Cost Savings**	**$1,043,296.01**
**Outpatient Costs for Intervention Patients**	
Number of Outpatient Visits per Patient-Year for Intervention Patients	12
Total Number of Outpatient Visits Per Team for Intervention Patients	4026
Average Medicaid Cost Per Outpatient Visit	$45
**Total Outpatient Costs for Intervention Patients**	**$182,981.70**
**Outpatient Costs for Control Patients**	
Number of Outpatient Visits per Patient-Year for Control Patients	11
Total Number of Outpatient Visits Per Team for Control Patients	3762
Average Medicaid Cost Per Outpatient Visit	$45
**Total Outpatient Costs for Control Patients**	**$170,982.90**
**Additional Outpatient Costs**	**$11,998.80**
**Total Estimated Medicaid Savings Per Year**	**$1,031,297.21**
**Return on Investment (Total Savings Divided by Expenses Per Team)**	**1.82**
Source: Authors' Analysis	
